# Externalizing Polygenic Liability, Brain Imaging Phenotypes, and Adolescent Substance Use Initiations: A Multistage Association and Mediation Analysis in ABCD

**DOI:** 10.64898/2026.04.08.717299

**Published:** 2026-04-12

**Authors:** Mengman Wei, Qian Peng

**Affiliations:** 1 Department of Neuroscience, The Scripps Research Institute, 10550 N Torrey Pines Rd, La Jolla, 92037, CA, U.S.

**Keywords:** Polygenic risk score, Externalizing behavior, Substance use initiation, Neuroimaging, Mediation analysis, ABCD Study

## Abstract

**Background:**

Externalizing liability is a strong risk factor for early substance initiation, but the neurobiological pathways linking polygenic risk to initiation remain incompletely characterized.

**Methods:**

Using the ABCD Study, we implemented a four-stage framework linking an externalizing polygenic risk score (extPRS) to baseline multimodal neuroimaging-derived phenotypes (IDPs) and longitudinal substance initiation (alcohol [primary], nicotine, cannabis, and any substance). First, we screened extPRS–IDP associations using covariate-adjusted linear models (age, sex, ancestry principal components, site/scanner variables; modality-specific covariates where applicable) and controlled multiple testing using false discovery rate (FDR) procedures. Second, we estimated direct extPRS associations with time-to-initiation using Cox proportional hazards models. Third, we fit joint Cox models including extPRS and each discovery-significant IDP, retaining outcome–IDP associations after within-outcome FDR correction. Fourth, we conducted mediation analyses for prioritized outcome–IDP pairs using an extPRS → IDP mediator model and an initiation model including both extPRS and IDP, estimating indirect (ACME) and direct (ADE) effects via bootstrap with multiple-testing control.

**Results:**

Among 10,608 participants, higher extPRS was associated with earlier initiation across outcomes, with the largest effects observed for nicotine and cannabis and a modest but significant effect for alcohol. Stage 1 identified thousands of extPRS-associated IDPs that were highly concordant across robustness specifications. Stage 3 prioritized outcome-specific IDPs associated with initiation beyond extPRS, with the number of retained IDPs varying across sensitivity settings (site-clustered vs. HC3 standard errors; SES covariates on/off) but showing a replicated core set across models. In Stage 4, mediation analyses showed that indirect effects of extPRS through IDPs were small in magnitude (ACME ≈ 10^−4^) and accounted for less than 2% of the total effect, while direct effects (ADE ≈ 0.02–0.05) remained strong across outcomes. FDR-significant mediation signals were observed only for alcohol and any-substance initiation, whereas no mediation effects survived multiple testing correction for cannabis or nicotine. Across outcomes, direct genetic effects were substantially larger than mediated effects, indicating that genetic liability operates primarily through direct pathways rather than through baseline brain measures.

**Conclusions:**

Externalizing polygenic liability is broadly associated with substance initiation, with robust and consistent direct effects across substances. Although specific frontal structural and microstructural phenotypes show statistically significant mediation signals, their contribution is small, suggesting that baseline brain measures explain only a limited proportion of genetic risk. This framework provides a scalable approach to prioritize neurobiological pathways linking genetic liability to early substance initiation while highlighting the dominant role of direct genetic effects.

## Introduction

Externalizing behaviors—including impulsivity, disinhibition, and related traits—are strongly associated with early substance use and later substance use disorders [1, 2]. Polygenic risk scores (PRS) for externalizing liability provide a quantitative measure of genetic susceptibility, but the pathways by which genetic liability translates into early initiation remain incompletely understood [3, 4]. Neurodevelopmental models suggest that frontal systems supporting inhibitory control and valuation may be particularly relevant during adolescence, yet large-scale studies linking externalizing PRS to neuroimaging markers and prospective initiation outcomes remain limited [5].

Neurodevelopmental frameworks further emphasize adolescence as a period of imbalance between heightened motivational drive and still-maturing cognitive control systems, with frontal circuitry supporting inhibitory control and valuation particularly implicated in risk behaviors [6, 7, 8]. However, prospective, multimodal studies that integrate externalizing PRS, neuroimaging phenotypes, and subsequent initiation outcomes at population scale remain relatively limited, despite rapid growth in ABCD-based research [9, 10, 11, 12].

Here, we present a multistage analytical framework linking externalizing polygenic liability to (i) neuroimaging phenotypes, (ii) time-to-initiation outcomes for alcohol and other substances, and (iii) mediation signals implicating specific brain phenotypes as potential intermediate pathways. Using the ABCD Study’s rich longitudinal and multimodal imaging data, we systematically screen imaging phenotypes, apply multiple-testing control, and prioritize interpretable brain markers that relate to initiation and partially mediate polygenic associations [9, 10, 11, 12, 13].

Our study aims are to: (i) identify neuroimaging phenotypes (IDPs) associated with externalizing polygenic risk; (ii) quantify direct associations between extPRS and substance initiation using regression-based models; (iii) test whether extPRS-associated IDPs predict initiation when modeled jointly with extPRS; and (iv) evaluate mediation (ACME, ADE, and proportion mediated) for prioritized outcome–IDP pairs.

## Methods

### Cohort and outcomes

We included ABCD Study^®^ participants with available genotype data sufficient to compute an externalizing polygenic risk score (EXT-PRS), at least one eligible neuroimaging phenotype, and longitudinal substance-initiation assessments. The primary outcome was alcohol initiation; secondary outcomes were initiation of nicotine, cannabis, and any substance.

Time-to-initiation was defined as the interval from baseline assessment to the first study visit at which the participant reported initiation of the relevant substance. Participants without initiation were censored at their last available follow-up visit. We constructed (time*,*event) variables for Cox proportional hazards models accordingly. EXT-PRS computation and substance-initiation phenotype harmonization followed previously described procedures [14, 15, 16, 17, 18, 19, 20], with key definitions summarized here and full implementation details provided in those reports.

### Externalizing polygenic risk score (EXT-PRS)

We derived an externalizing polygenic risk score (EXT-PRS) using externalizing GWAS summary statistics and ABCD genotype data after standard quality control. Summary statistics were harmonized to the ABCD reference alleles and genomic build as needed, and the PRS was computed using our previously published pipeline [14, 15, 16, 17, 18, 19, 20]. The EXT-PRS was *z*-standardized (mean = 0, SD = 1) prior to regression analyses.

### Neuroimaging phenotypes (IDPs)

We analyzed baseline multimodal neuroimaging-derived phenotypes (IDPs) from the ABCD core imaging tables, including structural MRI (e.g., cortical thickness, surface area, regional volumes, and intensity/contrast metrics), diffusion MRI measures (DTI and restriction spectrum imaging [RSI] summaries), and resting-state fMRI summary measures (e.g., connectivity- and variance-related metrics).

IDPs were extracted at the region-of-interest level across multiple parcellation and segmentation schemes (e.g., aseg and cortical atlas variants). For each table, we retained one baseline record per participant and standardized each IDP (*z*-score) within the analytic sample to improve comparability across modalities and scales.

### Covariates

We examined associations between EXT-PRS and IDPs using standardized linear regression. For each IDP, we fit a linear model with the IDP as the dependent variable and EXT-PRS as the main predictor, adjusting for key demographic and imaging covariates: age, sex, ancestry principal components (PCs), imaging site, scanner manufacturer, and scanner device serial number.

Covariate sets were modality-specific where appropriate. For structural MRI phenotypes, we additionally adjusted for intracranial volume (ICV) and, when applicable, global mean cortical thickness or total surface area. For diffusion MRI and fMRI measures, we further included motion and data-quality indices to account for acquisition-related artifacts.

Covariates were harmonized across modalities, with categorical variables one-hot encoded and all continuous variables winsorized at the 0.5% tails. Models were estimated using ordinary least squares (OLS) in statsmodels. Robust standard errors were computed, with clustering by site when specified. Associations were summarized using standardized β coefficients, standard errors, *p*-values, and partial $R^{2}$ values, where partial $R^{2}$ represents the variance uniquely explained by EXT-PRS.

To control for multiple testing across IDPs, we applied both Benjamini–Hochberg false discovery rate (FDR) [21] and Bonferroni correction [22]. In addition, we estimated the effective number of independent tests ($M_{\text{eff}}$) using the Li and Ji method [23] to account for correlation among IDPs. FDR-significant results after Li–Ji correction were reported separately for each model specification, including site-clustered and SES-adjusted sensitivity analyses.

### Externalizing polygenic risk score (extPRS), neuroimaging phenotypes (IDPs), and substance use initiation: multistage analysis pipeline

We implemented a four-stage analysis pipeline to connect externalizing polygenic liability (extPRS) with baseline neuroimaging-derived phenotypes (IDPs) and time-to-initiation of substance use (alcohol, nicotine, cannabis, and any substance). In brief, we (i) screened extPRS–IDP associations across all IDPs, (ii) tested the direct association of extPRS with initiation using Cox models, (iii) fit joint extPRS + IDP Cox models to prioritize IDPs associated with initiation beyond extPRS, and (iv) evaluated mediation for prioritized extPRS–IDP–initiation candidate pairs.

#### Stage 1: extPRS → IDP association screening (IDP-by-IDP linear regression)

##### Data inputs and preprocessing

The extPRS was computed and standardized to a *z*-score within the analytic sample. Baseline IDPs were loaded from a single harmonized IDP table, and covariates were loaded from a precomputed covariate table.

By default, we analyzed all numeric IDP columns, excluding non-phenotype fields (e.g., unnamed columns). For each IDP, we merged IDP values with extPRS and covariates, removed observations with missing values for model variables, and applied two-sided winsorization to the IDP outcome (default: 0.5% in each tail) to reduce the influence of extreme values. Each IDP was then standardized to a *z*-score within the analytic subset used for that model. IDPs with zero or near-zero variance after preprocessing were excluded.

##### Covariates

All Stage 1 models included the following covariates when available: age, sex, genetic ancestry principal components (PC1–PC20), site (modeled using one-hot encoding with k-1 dummy variables), scanner manufacturer (one-hot encoded), and scanner device serial number (one-hot encoded).

To better control modality-specific confounding, we applied modality-aware covariate rules based on the IDP name:

- For non-T1 modalities (e.g., diffusion MRI or fMRI; identified by keywords such as dti, dmri, fmri, rsi), we additionally included a motion covariate when available.
- For structural surface area or volume measures (identified by keywords such as area, vol, volume), we additionally adjusted for intracranial volume (ICV) to account for head size.

### Statistical model

For each IDP, we fit an ordinary least squares regression:

$${IDP}_{z}=\beta_{0}+\beta_{1}\cdot {extPRS}_{z}+\sum_{k}\gamma_{k}C_{k}+\varepsilon ,$$

where $C_{k}$ denotes the covariates defined above. For each IDP, we recorded the sample size, $\beta_{1}$, robust site-clustered and HC3 standard errors, *p*-values, model $R^{2}$, and partial $R^{2}$ for extPRS.

### Multiple-testing control

Across all tested IDPs, we controlled for multiple comparisons using (i) Benjamini–Hochberg false discovery rate (BH-FDR) across IDPs and (ii) Bonferroni correction across IDPs.

In addition, to account for correlation among IDPs when defining the discovery “hit” set used in later stages, we applied an effective number of independent tests approach [23]. The resulting discovery-significant IDPs were carried forward to Stage 3.

#### Stage 2: extPRS → initiation (direct effect; Cox proportional hazards models)

##### Outcomes and survival setup

For each initiation outcome (alcohol, nicotine, cannabis, and any substance), we modeled time-to-initiation using a Cox proportional hazards framework, where *time* denotes the follow-up time to initiation or censoring, and *event* indicates initiation (1 = initiated, 0 = censored).

##### Model specification

We fit:

$$Surv\left(\text{time},\;\text{event}\right)∼{extPRS}_{z}+\sum_{k}\gamma_{k}C_{k},$$

using Efron’s method for ties. Covariates matched the baseline set used in Stage 1 (age, sex, ancestry principal components, and available site/scanner indicators). We used site-clustered standard errors. Results are reported as hazard ratios (HRs) with 95% confidence intervals for extPRS.

#### Stage 3: extPRS + IDP → initiation (joint Cox models; candidate IDP prioritization)

##### Candidate IDPs

We restricted Stage 3 to the discovery-significant IDPs identified in Stage 1 after multiple-testing control, including correlation-aware adjustment.

##### Joint Cox models

For each outcome and each candidate IDP, we fit:

$$Surv\left(\text{time}\;,\;\text{event}\right)∼extPRSz+{IDP}_{z}+\sum_{k}\gamma_{k}C_{k},$$

again using Efron’s method for ties and site-clustered standard errors when available. Both extPRS and the IDP were re-standardized within the analytic subset for each model (after missing-data filtering), such that effect sizes correspond to a one standard deviation increase.

##### Multiple-testing control in Stage 3

Within each outcome, we applied Benjamini–Hochberg false discovery rate (BH-FDR) correction across candidate IDPs separately for each term (i.e., one correction for the extPRS term and one correction for the IDP term). Outcome–IDP pairs were retained as mediation candidates when the ${IDP}_{z}$ term satisfied FDR < 0.05.

#### Stage 4: Mediation analysis (ACME/ADE; bootstrap inference)

##### Candidate pairs and model forms

For each outcome–IDP pair passing Stage 3, we evaluated whether the IDP statistically mediated the association between extPRS and initiation. We treated ${extPRS}_{z}$ as the exposure and ${IDP}_{z}$ as the mediator, using:

- Mediator model (linear):
$${IDP}_{z}∼{extPRS}_{z}+\sum_{k}\gamma_{k}C_{k}$$
- Outcome model (logistic):
$$\;\text{event}\;∼{extPRS}_{z}+{IDP}_{z}+\sum_{k}\gamma_{k}C_{k}$$
  where *event* is the binary initiation indicator (consistent with the implementation used for mediation estimation). Covariates matched those used in earlier stages (age, sex, ancestry principal components, and available site/scanner indicators). extPRS and IDP were standardized within the mediation sample.

##### Quality control thresholds

To reduce unstable estimates, we required:

- at least 200 participants per outcome–IDP mediation analysis,
- at least 20 events and 20 non-events, and
- non-negligible variance in ${extPRS}_{z}$ and ${IDP}_{z}$.

##### Estimation and multiple-testing correction

We estimated the average causal mediation effect (ACME), average direct effect (ADE), and proportion mediated using nonparametric bootstrap inference with a target of 5,000 simulations. If bootstrap inference failed to converge, we used a pre-specified fallback sequence (bootstrap with 1,000 simulations; if needed, quasi-Bayesian simulation with 1,000 draws).

Finally, within each outcome we applied BH-FDR correction to mediation *p*-values (for ACME and proportion mediated).

## Results

### Sample characteristics

The analytic cohort included 10,608 ABCD participants with genotype data sufficient to compute the externalizing polygenic risk score (EXT-PRS), baseline neuroimaging phenotypes, and required covariates, and thus contributed to all initiation analyses.

Baseline age was 9.48 years (SD = 0.51; median = 9, IQR = 1). The sex distribution was 5,078 male (47.9%), 4,650 female (43.8%), and 880 unknown (8.3%). Ancestry composition was 5,710 White (53.8%), 2,077 Hispanic (19.6%), 1,480 Black (14.0%), 1,116 Other (10.5%), and 225 Asian (2.1%).

During follow-up, initiation events were observed for alcohol (n=3,964;37.4%), nicotine (n=591;5.6%), cannabis (n=378;3.6%), and any substance (n=4,302;40.6%), with remaining participants censored at their last available visit.

### Stage 1: extPRS → IDP association screening (IDP-by-IDP linear regression)

Using our primary specification (site-clustered standard errors with SES covariates included), we identified 3,307 IDPs significantly associated with extPRS (Li–Ji-adjusted BH-FDR q≤0.05). Sensitivity analyses showed robust findings across alternative specifications, including heteroskedasticity-robust (HC3) standard errors and exclusion of SES covariates. Specifically, we identified 5,460 hits for HC3 with SES included, 5,419 for site-clustered SE without SES, and 4,641 for HC3 without SES.

Despite differences in total discoveries, signals were highly stable across specifications: 3,184 IDPs were significant in both SES-on models, and 3,098 IDPs remained significant across all four settings (SES on/off × site-clustered/HC3). Effect sizes were highly concordant across overlapping IDPs (Pearson r≈0.995; Spearman ρ≈0.98), indicating strong robustness to model specification. Detailed results are provided in Supplementary Tables S1–S5.

Among the 3,098 robust IDPs, effects showed clear modality-specific patterns. Median associations were most negative for resting-state fMRI phenotypes (median β=-0.0240,n=122) and diffusion-derived phenotypes (dMRI median β=-0.0175, n=1,488; DTI-derived median β=-0.0207, n=305), with particularly strong negative shifts observed for rs-fMRI network metrics and DTI radial diffusivity. In contrast, structural MRI phenotypes showed modest positive-skewed effects (sMRI median β=0.0095, n=299; sMRI-derived median β=0.0130,n=321).

These patterns were consistent across parcellation schemes, hemispheres (LH median β=-0.0157, RH −0.0163), and global measures, arguing against hemisphere-specific artifacts. The strongest individual associations reached |β|≈0.054 SD per 1 SD increase in extPRS. Full IDP-level results and group summaries are provided in Supplementary Tables S1–S5.

### Stage 2: extPRS → initiation (direct effect; Cox proportional hazards models)

In Cox proportional hazards models adjusted for baseline covariates (age, sex, ancestry principal components, and site/scanner indicators), higher extPRS was associated with increased risk of substance initiation across all outcomes (n=10,599).

Under the site-clustered standard error specification without SES covariates, each 1 SD increase in extPRS was associated with increased hazards of initiation for alcohol (HR = 1.129, 95% CI 1.076–1.185, $p=7.1\times 10^{-7}$), nicotine (HR = 1.628, 95% CI 1.473–1.799, $p=1.16\times 10^{-21}$), cannabis (HR = 1.665, 95% CI 1.447–1.917, $p=1.11\times 10^{-12}$), and any substance (HR = 1.146, 95% CI 1.094–1.201, $p=1.18\times 10^{-8}$).

Results were robust to alternative variance estimators (HC3) and inclusion of SES covariates, with all hazard ratios remaining greater than 1 and statistically significant. For example, with SES adjustment, hazard ratios were 1.152 (alcohol), 1.562 (nicotine), 1.580 (cannabis), and 1.163 (any substance). These findings indicate that higher externalizing polygenic liability is associated with earlier initiation risk across substances, with the strongest effects observed for nicotine and cannabis. Full results are provided in Supplementary Tables S6–S9.

### Stage 3: extPRS + IDP → initiation (joint Cox models; candidate IDP prioritization)

In Stage 3, we evaluated discovery-significant IDPs from Stage 1 in outcome-specific joint Cox models including both extPRS and a single standardized IDP, and retained associations passing BH-FDR (q<0.05) on the IDP term.

The number of significant IDPs varied substantially across robustness specifications (site-clustered vs. HC3 standard errors; SES covariates on vs. off). Under site-clustered SE without SES covariates, we identified 173 (alcohol), 137 (any substance), 309 (cannabis), and 930 (nicotine) significant IDPs. With SES adjustment, these counts decreased to 31, 32, 137, and 459, respectively.

Overlap analyses indicated moderate replication for alcohol and any substance (Jaccard indices of 0.45 and 0.39, corresponding to 87 and 69 overlapping IDPs), but weaker replication for nicotine (Jaccard = 0.17). Notably, SES-adjusted models largely identified subsets of the non-SES-adjusted results (e.g., 30/31 for alcohol, 31/32 for any substance, 136/137 for cannabis, and ~451/459 for nicotine), suggesting that SES adjustment primarily reduced statistical power rather than introducing novel signals.

Across all specifications, we identified 1,630 unique outcome–IDP associations, of which 819 (~50%) replicated in at least two models. Several IDPs were consistently identified across all four specifications, suggesting a core set of robust neuroimaging correlates of substance initiation beyond extPRS.

### Stage 4: Mediation analysis (ACME/ADE; bootstrap inference)

We evaluated whether brain imaging-derived phenotypes (IDPs) mediate the association between polygenic risk scores (PRS) and substance initiation using bootstrap-based mediation models (5,000 simulations), adjusting for site clustering and socioeconomic status (SES).

Across all outcomes, the direct effect of PRS on initiation (ADE) was strong and highly significant (ADE ≈ 0.04, p<0.001). In contrast, indirect effects through brain IDPs (ACME) were small in magnitude (approximately −3 × 10^−4^ to −6 × 10^−4^), although some reached statistical significance. The proportion mediated was consistently low (< 2%), indicating that only a small fraction of genetic risk is transmitted through these brain measures.

Overall, these results indicate that genetic liability to substance initiation operates predominantly through direct pathways, with brain IDPs contributing only minor effects.

#### Outcome-specific mediation results

In the primary model (clustered standard errors with SES adjustment), we observed:

- **Alcohol initiation:** Six IDPs showed significant mediation after multiple testing correction (FDR < 0.05). ACME values ranged from −0.00071 to 0.00057, while ADE ranged from 0.0389 to 0.0415. The proportion mediated ranged from −1.77% to 1.44%.
- **Any-substance initiation:** Eleven IDPs remained significant after FDR correction. ACME ranged from −0.00070 to 0.00061, and ADE ranged from 0.0452 to 0.0467. The proportion mediated ranged from −1.53% to 1.31%.
- **Cannabis initiation:** No mediation effects survived multiple testing correction. Nominally significant ACME values ranged from −0.00016 to 0.00019, with ADE between 0.0180 and 0.0202.
- **Nicotine initiation:** No mediation effects survived multiple testing correction. Nominal ACME values ranged from −0.00019 to 0.00027, with ADE between 0.0283 and 0.0300.

These results indicate that robust mediation is present only for alcohol and any-substance initiation, and even in these outcomes, effect sizes are small.

#### Comparison of direct and mediated effects

Across all outcomes, ADE was substantially larger than ACME. ACME estimates were on the order of 10^−4^ and accounted for less than 2% of the total effect.

**Figure 1:**
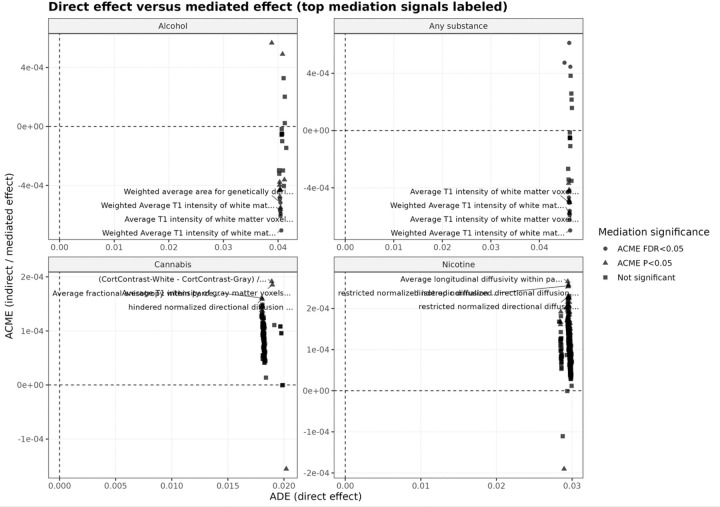
Direct versus mediated effects of externalizing polygenic risk across substance initiation outcomes. Scatter plots show the relationship between the direct effect (ADE; x-axis) and indirect (mediated) effect (ACME; y-axis) for each outcome (alcohol, any substance, cannabis, nicotine). Each point represents a neuroimaging-derived phenotype (IDP). Points are colored by mediation significance (ACME FDR < 0.05, ACME p<0.05, or not significant). Across all outcomes, ADE is substantially larger than ACME, with ACME values close to zero (on the order of 10^−4^). This separation indicates that genetic liability primarily influences substance initiation through direct pathways rather than through baseline brain imaging measures. Selected top IDPs are labeled for interpretability.

Visualization of ADE versus ACME (Figure 1) shows a clear separation between large direct effects and minimal mediated effects. This pattern indicates that genetic risk primarily influences substance initiation through direct pathways rather than through baseline brain structure or function.

#### Direction and interpretation of mediation effects

**Figure 2:**
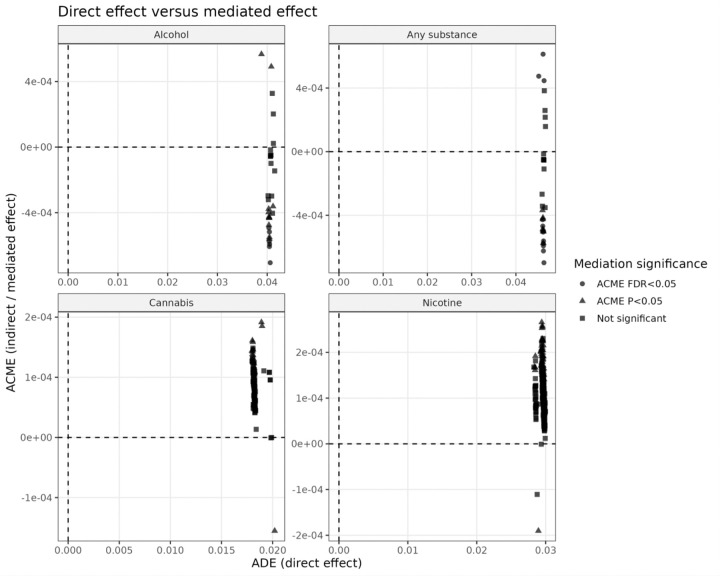
Direction and distribution of mediation effects across outcomes. Scatter plots of ADE (direct effect; x-axis) versus ACME (mediated effect; y-axis) for each outcome without labeling individual IDPs. Alcohol initiation shows predominantly negative ACME values, indicating a consistent buffering (attenuating) effect of brain features on genetic risk. Any-substance initiation shows both positive and negative ACME values, suggesting a mixture of risk-enhancing and protective pathways. Cannabis and nicotine show small and non-significant mediation effects, indicating weak and non-robust mediation patterns.

The direction of mediation effects differed by outcome (Figure 2):

- **Alcohol initiation:** All significant ACME estimates were negative, indicating that these brain features attenuate the effect of genetic risk, consistent with a buffering or compensatory role.
- **Any-substance initiation:** Both positive and negative mediation effects were observed, indicating the presence of both risk-enhancing and protective pathways.
- **Cannabis and nicotine initiation:** Mediation effects were small and did not survive multiple testing correction, suggesting weak and non-robust mediation.

Across outcomes, the strongest signals involved overlapping brain regions and similar classes of features, including structural measures and diffusion-based metrics, suggesting shared neurodevelopmental pathways.

#### Proportion mediated

**Figure 3:**
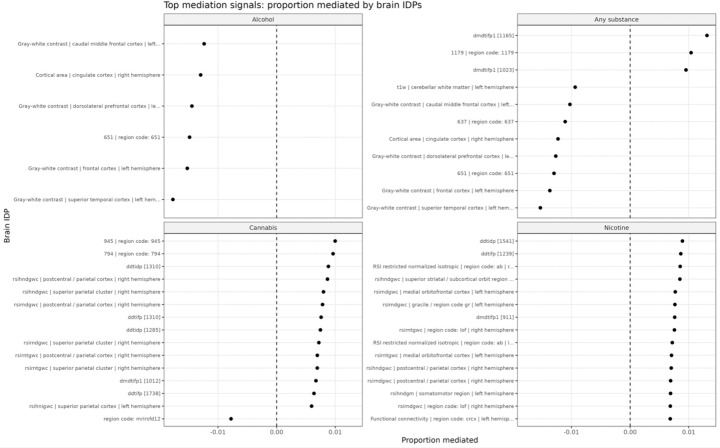
Proportion of genetic risk mediated by brain imaging-derived phenotypes. Top IDPs ranked by proportion mediated for each outcome. Across all outcomes, the absolute proportion mediated is small (generally < 2%). Alcohol initiation shows consistently negative mediation effects, indicating a buffering pattern. Any-substance initiation shows mixed positive and negative effects, while cannabis and nicotine show small and non-significant mediation. These results indicate that baseline brain features explain only a minimal proportion of genetic liability.

Figure 3 summarizes the proportion of genetic risk mediated through brain IDPs. Across all outcomes, the absolute proportion mediated was consistently below 2%:

- **Alcohol:** uniformly negative mediation effects (buffering pattern)
- **Any substance:** mixed positive and negative effects
- **Cannabis and nicotine:** small, non-significant effects

These findings indicate that baseline brain features explain only a minimal proportion of genetic liability.

#### Sensitivity analysis

To assess robustness, we repeated the mediation analysis using heteroskedasticity-consistent (HC3) standard errors while retaining SES adjustment.

The results were consistent with the primary model:

- ADE remained substantially larger than ACME
- Proportion mediated remained below 2%
- Alcohol showed consistently negative mediation effects
- Any substance showed mixed positive and negative effects
- Cannabis and nicotine remained non-significant

Although the specific IDPs identified varied slightly, similar classes of brain features, particularly white matter intensity and diffusion-based measures, were consistently implicated. These results indicate that the observed mediation patterns are robust to alternative variance estimation approaches.

## Discussion

In this study, we developed and applied a multistage framework to link externalizing polygenic liability (extPRS) with baseline neuroimaging phenotypes and prospective substance initiation in the ABCD cohort. Across all stages, we observed a consistent pattern: extPRS is broadly associated with both brain phenotypes and initiation risk, but mediation through baseline brain measures is limited.

### Principal findings

First, extPRS showed widespread associations with neuroimaging phenotypes. Thousands of IDPs were associated with extPRS, and these signals were highly consistent across model specifications. Effect sizes were strongly correlated across sensitivity settings, indicating that the extPRS–IDP associations are stable and not driven by specific modeling choices. These associations showed structured patterns across modalities, with negative shifts in diffusion and functional measures and more modest positive shifts in structural measures.

Second, extPRS was consistently associated with earlier substance initiation across all outcomes. Effect sizes were largest for nicotine and cannabis and more modest for alcohol and any substance, but all associations were statistically significant and robust across variance estimators and SES adjustment. These findings confirm that externalizing genetic liability is a broad risk factor for early substance initiation.

Third, joint models identified a large number of IDPs associated with initiation beyond extPRS. The number of identified IDPs varied across robustness specifications, but overlap analyses revealed a replicated core set of features. Adjustment for SES reduced the number of detectable associations but did not introduce new signals, suggesting that SES primarily affects statistical power rather than altering the underlying relationships. Across models, structural and diffusion-related features were consistently implicated.

Fourth, mediation analyses showed that only a small proportion of the extPRS effect on initiation is mediated through baseline brain phenotypes. Although several IDPs showed statistically significant indirect effects for alcohol and any-substance initiation, these effects were very small in magnitude (ACME on the order of 10^−4^), corresponding to less than 2% of the total effect. For cannabis and nicotine, no mediation effects survived multiple testing correction. Across all outcomes, the direct genetic effect (ADE) was substantially larger than the mediated effect, indicating that genetic liability operates primarily through direct pathways.

### Interpretation

Taken together, these findings suggest that externalizing genetic liability influences substance initiation through multiple pathways, but baseline brain structure and function capture only a small portion of this process.

The widespread extPRS–IDP associations indicate that genetic liability is reflected broadly across brain systems. However, the limited mediation effects suggest that these baseline brain measures are not the primary mechanism linking genetic risk to initiation behavior. Instead, they may represent downstream correlates or parallel processes rather than causal intermediates.

The consistent negative mediation effects observed for alcohol initiation suggest a buffering or compensatory role of certain brain features, particularly structural and white matter measures. In contrast, the mixed positive and negative effects observed for any-substance initiation indicate that both risk-enhancing and protective pathways may be present. The absence of robust mediation for cannabis and nicotine, despite strong direct genetic effects, further supports the conclusion that non-brain pathways or dynamic processes may play a larger role for these outcomes.

One possible explanation is that baseline imaging measures capture only a static snapshot of neurodevelopment, whereas substance initiation is influenced by dynamic processes over time, including environmental exposures, behavioral trajectories, and gene–environment interactions. In this context, the observed IDPs may reflect general neurodevelopmental variation associated with genetic liability rather than specific mediators of initiation risk.

### Methodological implications

This study demonstrates the value of a multistage framework that combines high-dimensional screening, survival modeling, and mediation analysis. By separating discovery (Stage 1), outcome association (Stage 3), and mediation (Stage 4), we reduce the risk of false positives and improve interpretability.

The robustness analyses further strengthen the findings. Results were consistent across different variance estimators and SES adjustment, and a substantial proportion of IDPs replicated across model specifications. This consistency suggests that the identified patterns are not artifacts of modeling choices.

At the same time, the large number of associations in Stage 1 and Stage 3 highlights the importance of careful multiple-testing control and prioritization strategies. The mediation step provides an additional layer of filtering, focusing attention on IDPs with potential mechanistic relevance.

### Limitations

Several limitations should be considered.

First, mediation analyses were based on baseline IDPs, which may not fully capture the dynamic neurodevelopmental processes that influence substance initiation. Longitudinal imaging data may provide stronger tests of mediation.

Second, although we adjusted for a comprehensive set of covariates, residual confounding cannot be ruled out, particularly for environmental and behavioral factors that were not explicitly modeled in the mediation framework.

Third, the mediation models used a simplified structure (extPRS → IDP → initiation), which may not capture more complex pathways involving multiple mediators or interactions.

Fourth, the effect sizes of the mediation signals were small and, although statistically significant in some cases, their practical impact is limited. These findings should therefore be interpreted as indicating limited mediation rather than strong mechanistic pathways.

Finally, the generalizability of the findings may depend on the characteristics of the ABCD cohort and the ancestry composition used in PRS construction.

### Future directions

Future work should extend this framework in several ways:

- Incorporate longitudinal imaging data to evaluate time-varying mediation effects
- Integrate environmental and behavioral factors to test gene–environment pathways
- Apply multivariate or network-based mediation models to capture more complex mechanisms
- Examine developmental trajectories rather than baseline measures alone

## Conclusions

We developed and applied a multistage framework linking externalizing polygenic risk to brain imaging phenotypes and prospective substance initiation in the ABCD cohort.

Across outcomes, extPRS was consistently associated with increased risk of substance initiation, with the strongest effects observed for nicotine and cannabis. ExtPRS was also broadly associated with multimodal neuroimaging phenotypes, indicating widespread neurobiological correlates of genetic liability.

However, mediation analyses showed that only a small proportion (< 2%) of the genetic effect is explained by baseline brain measures. Direct genetic effects were substantially larger than mediated effects across all outcomes, indicating that genetic liability operates primarily through direct or non-brain pathways.

Robust mediation signals were observed only for alcohol and any-substance initiation, and even in these cases, effect sizes were small. The identified brain features may play a minor or compensatory role rather than serving as primary mechanisms.

Overall, these findings suggest that while brain phenotypes are associated with genetic liability, they explain only a limited portion of its effect on early substance initiation. This framework provides a scalable and reproducible approach for integrating genetic, neuroimaging, and longitudinal outcome data to prioritize candidate pathways for further investigation.

## Figures and Tables

**Table 1:** Stage 3: Joint Cox models (extPRS + IDP)—number of FDR-significant IDPs by model and outcome. Number of brain imaging-derived phenotypes (IDPs) significantly associated with substance initiation in joint Cox models including externalizing polygenic risk scores (extPRS), after false discovery rate (FDR) correction. Results are shown across two modeling strategies (cluster-robust and HC3 variance estimators) with and without adjustment for socioeconomic status (SES).

Model group	SES adjustment	Alcohol	Any substance	Cannabis	Nicotine

cluster	Off	173	137	309	930
cluster	On	31	32	137	459
hc3	Off	106	107	0	163
hc3	On	6	47	0	13

## Data Availability

The analysis code and scripts used in this study are freely available at the following GitHub repository: https://github.com/mw742/ExtPRS-Brain-SUD. This study uses data from the Adolescent Brain Cognitive Development (ABCD) Study (https://abcdstudy.org), held in the NIMH Data Archive (NDA). The ABCD data version used was 5.1. The study is supported by the National Institutes of Health (NIH) and additional federal partners under multiple award numbers, including U01DA041048 and U01DA050987. A full list of funders is available at https://abcdstudy.org/federal-partners.html.
